# Lessons learned from the development of a school age food allergy education program

**DOI:** 10.1186/1710-1492-10-S2-A12

**Published:** 2014-12-18

**Authors:** Cathy A Gillespie, Nancy L Ross, Allan B Becker

**Affiliations:** 1 Children’s Allergy and Asthma Education Centre, Health Sciences Centre, Winnipeg, Manitoba, Canada; 2 University of Manitoba, Winnipeg, Manitoba, Canada

## Background

Education is required to effectively manage food allergy. In young children, parents are the primary managers. Once children enter school, a shift should begin towards a shared care model with children beginning to take on more responsibility. We previously developed a food allergy education program for parents of pre-school children and now have developed a new program for school age children and their parents building on our experience with the pre-school program.

## Method

Development steps included: synthesizing knowledge gained from expert advice, a literature review and parents; creation of program objectives, content, teaching tools and facilitator notes; sharing a program draft with experts for feedback; piloting the revised program; making further revisions during the pilot.

## Results

The pilot phase included 6 groups and 22 families (37 parents, 23 children). Children liked the activity-based sessions and benefited from meeting with peers. Parents appreciated the learning opportunity for children. Some parents suggested a less structured format for the adult sessions. Many parents highly valued the peer-to-peer discussions. Parents were interested in the practical aspects of dealing with other people, educating their child, avoiding risk and recognizing and treating reactions.

## Conclusions

In our experience, finding the right approach to food allergy education for school age parents is more complex than for parents of young children or even asthma education for school age parents. Most school age parents have lived with food allergy for a few years and have basic knowledge. However, at this stage their child’s expanding world and relationships present new issues for which many parents value practical advice from others living with the experience.

Health care providers should consider the importance of both professional and peer input into education for this population to ensure parents have correct current knowledge and practical skills to help them support and educate their child.

